# Factors contributing to clinically important health utility gains in cochlear implant recipients

**DOI:** 10.1007/s00405-020-06589-1

**Published:** 2021-01-15

**Authors:** Lida Müller, Petra Graham, Jasmin Kaur, Josie Wyss, Paula Greenham, Chris J. James

**Affiliations:** 1 Cochlear Implant Unit, Tygerberg Hospital, 5th Floor Gold Ave, Francie van Zijl Drive, Tygerberg, 7505 South Africa; 2 Department of Mathematics and Statistics, Macquarie University, North Ryde, NSW 2109 Australia; 3 Cochlear AG, Peter Merian-Weg 4, 4052 Basel, Switzerland; 4 Greenham Research Consulting Ltd, Downland House, Ashbury, SN6 8LP UK; 5 Cochlear France SAS, 135 Route de Saint Simon, 31100 Toulouse, France

**Keywords:** Quality of life, Screening, Cochlear implant, Hearing loss, Predicting outcomes, Cost-effectiveness

## Abstract

**Purpose:**

Cochlear implantation can restore access to sound and speech understanding in subjects with substantial hearing loss. The Health Utilities Index Mark III (HUI3) measures the impact of an intervention on the patient’s quality of life and is sensitive to changes in hearing. In the current study we used factor analysis to predict a clinically important gain in HUI3 scores in adult cochlear implant recipients.

**Methods:**

Data were collected in an observational study for 137 adult recipients from a single center who had at least 1-year HUI3 follow-up. Demographic and other baseline parameters were retrospectively analyzed for their association with a clinically important HUI3 scale gain, defined as at least 0.1 points. Data were also collected for the speech spatial qualities (SSQ) scale.

**Results:**

Baseline telephone use and HUI3 hearing, speech and emotion attribute levels were significantly associated with clinically important gains in HUI3 scores. However, SSQ scores increased significantly with or without clinically important HUI3 gains.

**Conclusion:**

Those subjects who were unhappy or experienced difficulties communicating with strangers or in a group were twice as likely to obtain a clinically important gain in health utility compared to those who were happy or had less difficulty communicating. Subjects who were unable to use the telephone prior to cochlear implantation were one and a half times more likely to obtain a clinically important gain. The SSQ scale was more sensitive to hearing improvements due to cochlear implantation. An inability to use the telephone is an easy to assess biomarker for candidacy for cochlear implantation.

## Introduction

The success of a healthcare intervention is often measured by its impact on the patient’s quality of life. This provides a tool for comparing the success of different treatments, regardless of the condition they are targeting [1]. Standardized measures of change in health-related quality of life are used to provide a health utility score. This can then be used to calculate any gains in quality-adjusted life years and assess a treatment’s cost-effectiveness [2, 3]. Three generic preference-based questionnaires are commonly used to provide a measure of health utility: EuroQol 5 dimensions (EQ-5D), Health Utility Index 3 (HUI3) and short-form 6 dimensions (SF-6D) [4–6]. Of these, only the HUI3 is sensitive to changes in hearing and is recommended as the questionnaire of choice for studies evaluating hearing treatments [7–9]. These generic measures are not as sensitive to perceived changes in hearing as many of the condition-specific measures available, e.g., the Speech Spatial Hearing questionnaire [10]. However, they provide a measure of the overall benefit to a recipient’s quality of life and are used in the cost-effectiveness analysis which informs many funding bodies who make decisions on healthcare provision [2, 11].

The HUI3 consists of 15 questions addressing eight individual health attributes: vision, hearing, speech, ambulation, dexterity, emotion, cognition and pain. Each health attribute has 5 or 6 levels and a weighted health utility score assigned to each level which varies by attribute. These scores are then combined to give a measure of the overall health utility [5]. Scores range from zero (dead) to one (perfect health) or even a negative score indicating a state worse than dead. HUI3 scores of less than 0.7 are considered to indicate a severe disability, between 0.7 and 0.88 a moderate disability, and 0.89 or better a mild or no disability [12]. An increase in score of at least 0.03 is thought to represent a noticeable improvement in quality of life for a patient [13] and an increase of 0.1, a minimum clinically important change [14]. In studies looking at the benefits of fitting hearing aids, gains in health utility ranged from 0.06 [15] to 0.12 [1, 16]. However, scores remain in the moderate to severe disability category even after treatment [12]. Cochlear implantation (CI) provides an alternative treatment for those with severe to profound hearing loss. In these cases, HUI3 scores following cochlear implantation using the HUI3 are consistently higher than with hearing aids, with gains of around 0.20 (range 0.05–0.4) [11, 17].

In South Africa approximately 83% of the population depends on public health and 17% on the private healthcare market financed by the medical industry. Funding is provided for CIs on an ad hoc basis from the public health sector through Tygerberg Hospital, with large waiting lists for adult candidates. In countries where access to CI is limited, establishing who will benefit most is one way of prioritizing treatment. For cochlear implantation, however, outcomes are difficult to predict from preoperative measures [18]. An actuarial approach acknowledges this variability in outcomes and expresses the a priori odds of getting a better result post-treatment [14, 19]. Up until now the definition of a ‘better result’ has been a greater improvement in speech perception outcomes. Improvements in quality of life, however, have consistently been shown to be independent of audiological performance and therefore may be more meaningful for patients [11].

There is little published work in this area. A few studies have identified the preoperative presence of comorbidities, dizziness or tinnitus and the duration of deafness and depression scores as factors that have a significant influence on the change in HUI3 scores resulting from implantation [18, 20–22].

The aim of this study was to determine which baseline factors may significantly influence clinically important gains in HUI3 in a large sample. A retrospective analysis was performed on data collected from one South African clinic who took part in the Cochlear Implant Recipient Observational Study (IROS) [17]. We considered a gain of ≥ 0.1 in HUI3 multi-attribute score as clinically important based on the literature (e.g., UKCISD, 2005) [14]. The intention was to provide a guide for clinicians when counseling potential patients about the benefits of a CI and to help them prioritize candidates in clinics with large waiting lists and limited resources. The limitations of the HUI3 and the factors that contribute to any gains are also explored, so that the health utility changes resulting from CIs can be better understood and put in context.

## Methods

Data were gathered via a voluntary online international observational registry for hearing implants initiated by Cochlear Ltd (Sydney, NSW, Australia). Data were collected in accordance with the Declaration of Helsinki. Ethics approval was obtained from the Health Research Ethics Committee 1, Stellenbosch University (reference N15/02/015). Each subject provided their written informed consent for their data to be included in the registry.

### Subjects

We extracted data for all adult unilateral CI recipients (> 17 years old) implanted at the Tygerberg Hospital, Stellenbosch University Cochlear Implant Unit, between 2012 and 2019. Baseline data were collected prior to activation of the processor after a decision to implant had been made. Follow-up data had been collected up to 3 years post-implantation in some cases. However, here we focus on the gain from baseline to 1-year post-activation. Self-assessments were completed by the recipient either online or on paper and then entered into the database by a representative of the clinic.

### Outcome measures

The Health Utilities Index Mark 3 (HUI3), a generic measure of health utility gains, was the primary measure [13]. Change from baseline to first follow-up in the HUI multi-attribute above or below 0.1 units was the primary outcome in this study.

Table 1 illustrates the structure of the HUI3 hearing attribute levels. Levels are designated based on the combined responses to two questions about hearing. Levels 1 and 2, as the highest/best levels, indicate that the respondent does not always need a hearing aid to hear what is said, whereas the lower levels of performance 3–5 require use of a hearing aid (or other assistive hearing device) and report the level of performance with that device. Level 6 indicates unable to hear anything at all, even with hearing aids. Furthermore, only levels 1–3 indicate that respondents can hear what is said in more difficult situations. We have designated the categories “able to hear” and “unable to hear” to the hearing attribute levels 1–3 and 4–6 for the purposes of the discussion. Obtaining level 3 (or greater) means that the intervention was essentially successful in restoring the capacity to hear what is said, even in more challenging situations, so long as a hearing aid, or in this case CI, is being used. For CI we would assume that this is the highest hearing attribute level that can be achieved in most cases for obvious reasons.

**Table 1:**
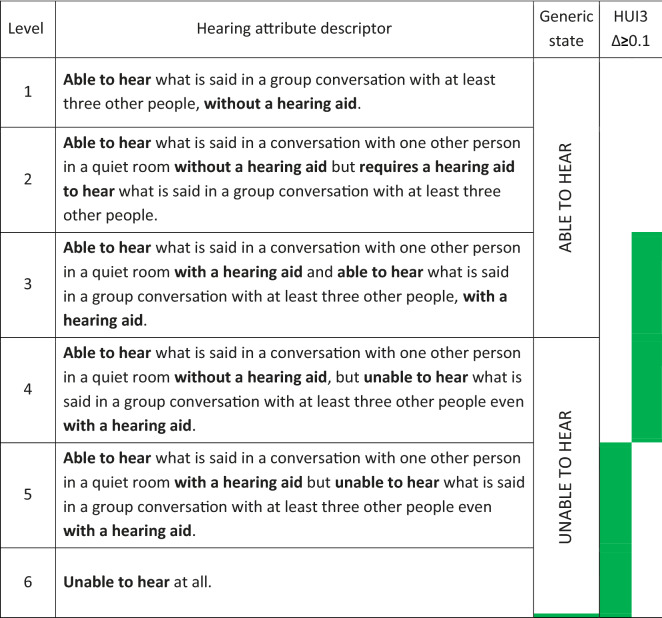
HUI3 hearing attribute levels and descriptors from Feeny et al. [5]

The third column indicates binary categories of “able to hear” or “unable to hear” that we define here. The final column indicates upward transitions in adjacent levels (shaded) that could result in ≥ 0.1 HUI3. All upward transitions of greater than one level potentially result in ≥ 0.1 HUI3 gain

Weighting scores for given levels of each health attribute are used to produce a HUI3 “total” or multi-attribute score combining all eight attributes. For most attributes at least two levels need to be gained to result in changes of ≥ 0.1 in the combined HUI3 multi-attribute scores. This is a property of the way the HUI3 was constructed. However, as shown in Table 1, some transitions between adjacent hearing attribute levels (e.g., for hearing 6–5, and 4–3) may result in changes of ≥ 0.1 in the combined HUI3 multi-attribute scores.

As a secondary outcome measure data were also collected for the speech spatial qualities (SSQ) scale [10]. This is a disease-specific scale which aims to evaluate the subject’s speech understanding in quiet and noise, spatial perception and the clarity, separation and identification of sounds. Additional information was collected about each subject’s hearing history and demographics. Changes of at least 1.0 unit on the SSQ subscales indicate a clinically relevant change [23].

### Statistics

Because interest was in those who gained at least the minimum clinically important difference (MCID) of 0.1 HUI3 multi-attribute score units, change in HUI3 multi-attribute score was dichotomized at this value with a 1 representing achievement of at least the MCID and 0 otherwise. Fisher’s exact test was used to determine the association between the dichotomized HUI3 multi-attribute change score and variables of interest. Table 3 shows the variables considered with variables summarized using count and percent. Levels for the eight attributes were dichotomized into worse/better outcomes so that counts in each category were sufficiently large to detect significant differences between proportions, if they existed. Paired *t* tests were used to examine change over time from baseline to year 1 follow-up. *p* values less than 0.05 were considered significant. All analyses were conducted in R^4^ [24].

## Results

### Gains in HUI3 scores

**Fig. 1:**
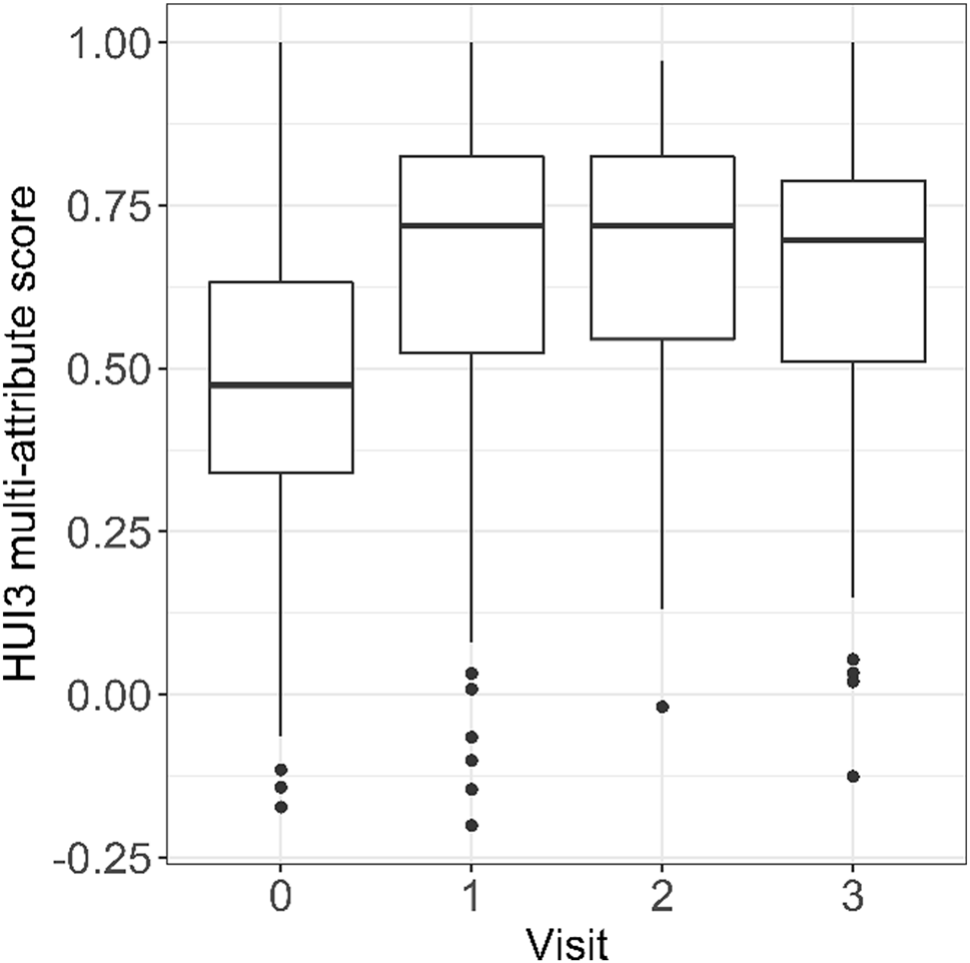
HUI3 multi-attribute scores at baseline and at 1, 2 and 3 years post-implant. Boxes show quartile values and the line the median value. Whiskers indicate the minimum and maximum value within 1.5 times the interquartile range and circles any outliers

One hundred and seventy-five subjects had baseline HUI data, 137 of those had follow-up at 1 year, 97 at year 2 and 75 at year 3. A statistically significant and clinically relevant improvement in HUI3 multi-attribute scores from baseline was seen for the group at year 1 (mean change = 0.16, 95% CI 0.11–0.21; *p* < 0.001), which was maintained at years 2 and 3 (Fig. 1, left). In total, 81/137 (59%) subjects had a ≥ 0.1 HUI3 gain at 1 year. As can be seen in the right panel of Fig. 1, preoperatively 65% were at generic “unable to hear” levels (4–6). Postoperatively this situation was more than reversed with 77% at generic “able to hear” levels (1–3).

A closer look at the individual changes in hearing attribute levels is available in Table 2, comparing preoperative with 1-year postoperative values for 137 subjects with both data points. Two contingencies contributed most often to ≥ 0.1 gain in HUI3 multi-attribute score; those at level 5 and 6 preoperatively, or generically “unable to hear”, moving to levels 1–3 postoperatively. Table 2 also indicates that about one-third (*n* = 46, 33.6%) of subjects had no change in hearing attribute level, with by far most of those starting and finishing at level 3. Overall, 74 (54%) subjects fell above the dividing line for significant hearing attribute level increase and 63 (46%) below. Thus, the transitions in hearing attribute level contributed to 74 of the total 81/137 subjects with ≥ 0.1 overall HUI3 gain.

**Table 2:**
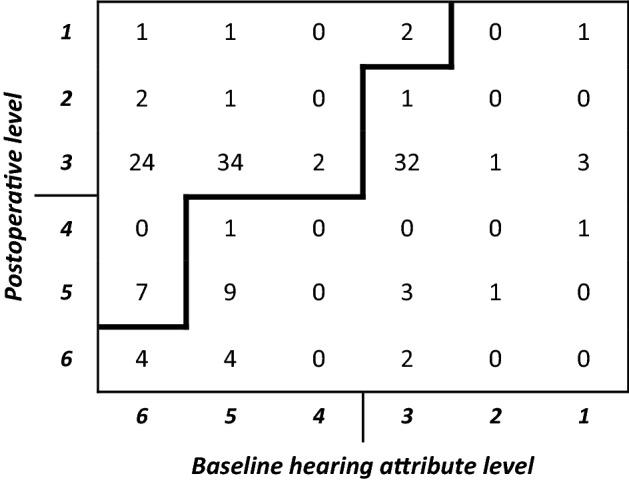
Cross-tabulation of the number of subjects at each hearing attribute level at baseline and postoperatively

Above the bold line indicates contingencies which would potentially result in ≥ 0.1 gain in HUI3 multi-attribute score as indicated in Table 1

### Baseline predictors of clinically important HUI3 gains

In Table 3 we present the results of the factor analysis of baseline variables on the bivariate outcome for less than or greater than a 0.1-point gain in HUI3 score. Fisher’s exact tests suggested that telephone use prior to implantation and the hearing, speech and emotion HUI3 attributes was associated with presence or absence of a clinically important gain from baseline to visit 1 (Table 3). It is obvious also that baseline hearing attribute level influenced clinically significant gains, as can be predicted by the way attribute scores are combined in the HUI3 (Table 1 and section).

**Table 3:** Count (percentage) of those who did or did not obtain a clinically important gain in HUI3 multi-attribute score (*p* value from Fisher’s exact test)

Categorical covariate at baseline	HUI gain < 0.1 *n* = 56 *n* (%)	HUI gain ≥ 0.1 *n* = 81 *n* (%)	*p* value
Female	37 (66.1)	52 (64.2)	0.857
Right-hand side implant	44 (78.6)	53 (65.4)	0.126
Implant side imaging indicates anomaly	6 (11.1)	6 (7.4)	0.542
Language			0.061
Afrikaans	18 (32.1)	16 (19.8)	
English	26 (46.4)	54 (66.7)	
Other	12 (21.4)	11 (13.6)	
Additional handicaps	3 (5.4)	1 (1.2)	0.305
Tinnitus present at baseline	26 (46.4)	44 (54.3)	0.389
Dizziness present at baseline	32 (57.1)	44 (54.3)	0.861
Onset of deafness			0.699
Progressive	39 (70.9)	60 (74.1)	
Sudden	16 (29.1)	21 (25.9)	
Hearing loss degree (implant side)			0.147
Profound	44 (78.6)	72 (88.9)	
Not profound	12 (21.4)	9 (11.1)	
Hearing loss degree (contralateral)			0.674
Profound	43 (76.8)	65 (80.2)	
Not profound	13 (23.2)	16 (19.8)	
Duration of deafness			0.481
≤ 13 years	11 (19.6)	26 (32.0)	
14–24 years	17 (30.3)	21 (25.9)	
25–37 years	15 (26.7)	18 (22.2)	
38+ years	12 (21.4)	16 (19.8)	
Hearing aid use in the contralateral ear at baseline	18 (32.1)	33 (40.7)	0.489
Age group			0.187
18–34	15 (26.8)	9 (11.1)	
35–44	8 (14.3)	16 (19.8)	
45–54	8 (14.3)	17 (21.0)	
55–64	8 (14.3)	15 (18.5)	
65–91	17 (30.4)	24 (29.6)	
**No telephone use**	**24 (42.9)**	**51 (63.0)**	**0.024**
*Etiology*			*0.560*
Congenital onset of deafness	15 (27.3)	16 (19.8)	0.405
Usefulness of implant side hearing aid			0.178
Marginally or not at all	28 (65.1)	48 (78.7)	
Moderately or more	15 (34.9)	13 (21.3)	
Usefulness of contralateral hearing aid			0.072
Marginally or not at all	17 (40.5)	35 (59.3)	
Moderately or more	25 (59.5)	24 (40.7)	
HUI3 attribute vision			1.000
Able to read with glasses or better	54 (96.4)	77 (95.1)	
Unable to read with glasses or worse	2 (3.6)	4 (4.9)	
HUI3 attribute hearing			**< 0.001**
Able to hear group with a hearing aid or better	**33 (58.9)**	**14 (17.3)**	
Unable to hear group with hearing aid or worse	**23 (41.1)**	**67 (82.7)**	
HUI3 attribute speech			**0.035**
Able to be understood partially by strangers or better	**49 (87.5)**	**58 (71.6)**	
Unable to be understood by strangers or worse	**7 (12.5)**	**23 (28.4)**	
HUI3 attribute ambulation			0.648
Able to walk neighborhood aided or unaided	55 (98.2)	77 (95.1)	
Walk short distances only or requires wheelchair	1 (1.8)	4 (4.9)	
HUI3 attribute dexterity			0.165
Limitations but independent or no limitations	54 (96.4)	81 (100)	
Not independent	2 (3.6)	1 (0)	
HUI3 attribute emotion			**0.030**
Somewhat happy or better	**50 (89.2)**	**59 (72.8)**	
Somewhat unhappy or worse	**6 (10.7)**	**22 (27.2)**	
HUI3 attribute cognition			0.590
Forgetful but thinks clearly or not forgetful	51 (91.1)	70 (86.4)	
Forgetful with difficulties or can’t remember at all	5 (8.9)	11 (13.6)	
HUI3 attribute pain			0.513
Mild or no pain; no activities prevented	47 (83.9)	63 (77.8)	
Moderate or more pain; activities prevented	9 (16.1)	18 (22.2)	

Items in italics represent unreliable figures due to very small counts in some categories. Items in bold were significant at the 5% level

Subjects unable to use the telephone preoperatively were one-and-a-half times more likely to obtain ≥ 0.1 gain in HUI3 scores (odds ratio OR 1.47); those reporting difficulty to be understood by strangers or worse twice as likely (OR 2.27); and those reporting being unhappy or worse two-and-a-half times more likely (OR 2.53). There was no effect of age, sex or duration of deafness. To put these into context, subjects unable to hear with hearing aids or worse at baseline had twice as much chance to obtain clinically important HUI3 gain as those who reported better hearing at baseline.

At baseline, subjects unable to hear were one-and-a-half times (OR 1.52) more likely not to use the telephone compared to those that did. Of participants who gained ≥ 0.1 in HUI3 score, 63% did not use the telephone at baseline, while among those who did not gain ≥ 0.1 in HUI3 score a significantly smaller proportion did not use the telephone at baseline. By visit 1, only 18/137 subjects were still not able to use the telephone, with eight of these in the ≥ 0.1 and ten in the < 0.1 gain groups.

### Gains in SSQ scores according to gain in HUI3 scores

SSQ scores improved significantly from baseline to 1 year both for who obtained clinically important gains in HUI3 score (mean SSQ change 3.18, 95% CI 2.73–3.62, *p* < 0.001) and for those who did not (mean SSQ change 2.43, 95% CI 1.98–2.88, *p* < 0.001).

## Discussion

Baseline scores placed the majority of the sample in the severe disability category of HUI3 multi-attribute score (Fig. 1) levels, i.e., where at least one attribute is at a reduced level of function that cannot be corrected and prevents many activities [12]. A score of around 0.45 is typical for a cohort of hearing-impaired individuals with sensorineural hearing loss and thus, even with a large increase in health utility, many implanted adults are expected to still score in the severely disabled range [1, 8, 11, 15, 17, 20].

After implantation, there was a significant gain in HUI3 scores at 1 year which was maintained at 2 and 3 years. At least 50% of the subjects had moved into the moderate disability category (Fig. 1), where at least one attribute is at a reduced level of function that cannot be corrected and/or prevents some activities. However, we see that most subjects reached level 3 on the hearing attribute scale. This level describes functional hearing with the use of a “hearing aid” (in this case a CI). The CI as an intervention in a deaf ear does not allow the subject to obtain hearing level 2 or 1 by their definition (Table 1), so this limits the gain that can be achieved. Levels 1–2 would generally result in a HUI3 score indicating mild or no disability in the absence of disabilities in other health attributes.

Some combination of other health attribute levels combined with a change in the hearing attribute may potentially attenuate the multi-attribute gain. Conversely, an improvement in hearing may have effect on other health attributes and thus increase the HUI3 gain. However, as indicated in Table 1, traversing to level 3 from levels 4–6 gives > 0.1 in HUI3 multi-attribute scores, as does from 6 to 5, and thus the change in the distribution of hearing attribute levels largely accounted for the overall group gains observed (Fig. 1).

Of the factors considered in the logistic regression analysis telephone use and the speech, hearing and emotion attributes on HUI3 were significant. Those who did not use the telephone at baseline were 1.5 times more likely to get a clinically meaningful gain on the HUI3 from implantation. As expected, subjects in the poor hearing category at baseline (hearing attribute levels 4, 5 or 6) were also more likely not to use the telephone (OR 1.52). Thus, telephone use is an easy parameter to use for the screening of potential adult CI candidates presenting to the audiology clinic. Take up of implants in adults is poor with less than 10% of suitable candidates receiving implants in western countries [25]. This is thought, in part, to be due to lack of referral to cochlear implant centers. With poor access to adult hearing screening in many countries, the capability to use the telephone provides a simple criterion for urgent referral for cochlear implant assessment. This approach is now supported by the results of this study.

The Fisher’s exact test showed that there was also a non-random association between a meaningful gain on the HUI3 and the hearing, speech and emotion single attributes. Not surprisingly, those subjects with poor baseline hearing were more likely to gain more than 0.1 points. This comes from changes in the hearing attribute scale where subjects move from the worst hearing levels to the better levels following implantation (Tables 1, 2). However, the study did highlight an anomaly with the HUI3 whereby a high proportion of subjects selected level 3 both before and after implant, thus showing no change on this measure. This highlights the need for more sensitive disease-specific measures of subjective benefit, such as the SSQ, to be used as well as generic quality of life questionnaires. The SSQ was still sensitive to changes in hearing brought about by CI, even in the group where there was no clinically important gain in HUI3. For that group the mean improvement of 2.43 on the SSQ scale was highly clinically significant.

Those who reported that strangers were unable to understand them, or even poorer verbal communication, were twice as likely to have a clinically significant gain in health utility. Improved overall verbal communication due to better hearing and other rehabilitation following CI may have contributed to this effect.

Most subjects in both groups reported being happy or somewhat happy prior to implantation. However, those who were unhappy prior to implant were twice as likely to show a meaningful gain in the HUI3, probably due to the implant and rehabilitation process improving their communication and thus emotional state. Significant hearing loss has long been associated with depression, and this is the one factor which has previously been identified as linked with change in HUI3 scores following implantation [20]. However, the number of subjects here was small so the results must be treated with caution.

In keeping with the larger cohort from the IROS study reported by Lenarz et al., there was no significant association between age or duration of deafness and the HUI3 outcomes [17]. Age should not be a barrier to receiving an implant, and quality of life benefits are not reduced in the elderly population [21, 22, 26]. The HUI3 result is contrary to speech perception-based outcome measures which consistently show an association between a short duration of deafness and better scores [18]. This reinforces the need to consider more holistic measures to assess the benefits of implantation than the standard measures currently used in the clinic (e.g., listening effort). The HUI is a standardized measure which allows comparison across healthcare treatments and thus has an important role to play in the overall evaluation of treatments. However, it has its limitations, for example beneficial practices such as early intervention act to reduce the impact of hearing loss on quality of life but decrease the health utility gain. If the benefits of implantation are to be truly represented, then more sensitive and clinically relevant measurement tools are needed.

## Limitations

This was an observational study, and enrolment bias may be present in the sample. Investigators were instructed to offer participation to all consecutively implanted adult and adolescent Nucleus^®^ implant recipients. However, recipients who were more motivated may have been more likely to agree to participate, which may influence outcomes. Data for some subjects were also included in the prior publication by Lenarz et al. [17]. As self-assessment was required, those with severe comorbidities and those who are unable to read were less likely to be included. Baseline evaluations were made between the day of the surgery and the first fitting, which may result in a recall bias. Baseline responses will have been influenced by the knowledge that recipients were getting an implant.

## Conclusions

After cochlear implantation there was a statistically significant group gain in HUI3 scores at 1 year which was maintained at years 2 and 3. Those who did not use the telephone at baseline were 1.5 times more likely to get a clinically important gain of ≥ 0.1 in HUI3 multi-attribute score from cochlear implantation. Those with lower hearing, speech or emotion HUI3 single-attribute levels prior to implantation were also more likely to obtain clinically important gains in HUI3 scores. There was no effect of age or duration of deafness. An incapacity to use the telephone may be a useful biomarker for cochlear implant candidacy prior to formal audiological evaluation.

For those the subjects who obtained < 0.1-point gain in HUI3, this could often be attributed to a lack of change in the hearing attribute level. A large proportion of this comes from the structure of the HUI3 hearing scales where subjects remain at level 3, able to hear with a hearing aid. However, mean hearing performance measured by the SSQ still increased significantly for that group after CI. The HUI3 scale stands in good stead as a health-utility instrument to measure the benefits of cochlear implantation for those with severe handicap due to hearing impairment, but lacks sensitivity to changes in hearing performance compared with a disease-specific measure such as the speech, spatial qualities (SSQ) scale.

## References

[1] Swan IR, Guy FH, Akeroyd MA (2012). Health-related quality of life before and after management in adults referred to otolaryngology: a prospective national study. ClinOtolaryngol.

[2] Foteff C, Kennedy S, Milton AH, Deger M, Payk F, Sanderson G (2016). Cost-utility analysis of cochlear implantation in Australian adults. OtolNeurotol.

[3] Saunders JE, Francis HW, Skarzynski PH (2016). Measuring success: cost-effectiveness and expanding access to cochlear implantation. OtolNeurotol.

[4] Herdman M, Gudex C, Lloyd A, Janssen M, Kind P, Parkin D, Bonsel G, Badia X (2011). Development and preliminary testing of the new five-level version of EQ-5D (EQ-5D-5L). Qual Life Res.

[5] Feeny D, Furlong W, Torrance GW, Goldsmith CH, Zhu Z, DePauw S, Denton M, Boyle M (2002). Multi-attribute and single-attribute utility functions for the health utilities index mark 3 system. Med Care.

[6] Brazier J, Roberts J, Deverill M (2002). The estimation of a preference-based measure of health from the SF-36. J Health Econ.

[7] Arnoldner C, Lin VY, Bresler R, Kaider A, Kuthubutheen J, Shipp D, Chen JM (2014). Quality of life in cochlear implantees: comparing utility values obtained through the Medical Outcome Study Short-Form Survey-6D and the Health Utility Index Mark 3. Laryngoscope.

[8] Summerfield AQ, Barton GR (2019). Sensitivity of EQ-5D-3L, HUI2, HUI3, and SF-6D to changes in speech reception and tinnitus associated with cochlear implantation. Qual Life Res.

[9] Yang Y, Longworth L, Brazier J (2013). An assessment of validity and responsiveness of generic measures of health-related quality of life in hearing impairment. Qual Life Res.

[10] Gatehouse S, Noble W (2004). The speech, spatial and qualities of hearing scale (SSQ). Int J Audiol.

[11] Crowson MG, Semenov YR, Tucci DL, Niparko JK (2017). Quality of life and cost-effectiveness of cochlear implants: a narrative review. AudiolNeurotol.

[12] Feng Y, Bernier J, McIntosh C, Orpana H (2009). Validation of disability categories derived from Health Utilities Index Mark 3 scores. Health Rep.

[13] Feeny D, Furlong W, Boyle M, Torrance GW (1995). Multi-attribute health status classification systems: Health Utilities Index. Pharmacoeconomics.

[14] UK Cochlear Implant Study Group (2004). Criteria of candidacy for unilateral cochlear implantation in postlingually deafened adults III: prospective evaluation of an actuarial approach to defining a criterion. Ear Hear.

[15] Barton GR, Bankart J, Davis AC, Summerfield QA (2004). Comparing utility scores before and after hearing-aid provision: results according to the EQ-5D, HUI3 and SF-6D. Appl Health Econ Health Policy.

[16] Grutters JP, Joore MA, van der Horst F, Verschuure H, Dreschler WA, Anteunis LJ (2007). Choosing between measures: comparison of EQ-5D, HUI2 and HUI3 in persons with hearing complaints. Qual Life Res.

[17] Lenarz T, Muller L, Czerniejewska-Wolska H, Vallés Varela H, OrúsDotú C, Durko M, HuarteIrujo A, Piszczatowski B, Zadrożniak M, Irwin C, Graham PL, Wyss J (2017). Patient-related benefits for adults with cochlear implantation: a multicultural longitudinal observational study. AudiolNeurootol.

[18] Lazard DS, Vincent C, Venail F, Van de Heyning P, Truy E, Sterkers O, Skarzynski PH, Skarzynski H, Schauwers K, O'Leary S, Mawman D, Maat B, Kleine-Punte A, Huber AM, Green K, Govaerts PJ, Fraysse B, Dowell R, Dillier N, Burke E, Beynon A, Bergeron F, Başkent D, Artières F, Blamey PJ (2012). Pre-, per- and postoperative factors affecting performance of postlinguistically deaf adults using cochlear implants: a new conceptual model over time. PLoS ONE.

[19] Lovett RE, Vickers DA, Summerfield AQ (2015). Bilateral cochlear implantation for hearing-impaired children: criterion of candidacy derived from an observational study. Ear Hear.

[20] Summerfield AQ, Marshall DH (1995). Preoperative predictors of outcomes from cochlear implantation in adults: performance and quality of life. Ann OtolRhinolLaryngolSuppl.

[21] Ramos A, Guerra-Jimenez G, Rodriguez C, Borkoski S, Falcon JC, Perez D (2013). Cochlear implants in adults over 60: a study of communicative benefits and the impact on quality of life. Cochlear Implants Int.

[22] Olze H, Gräbel S, Förster U, Zirke N, Huhnd LE, Haupt H, Mazurek B (2012). Elderly patients benefit from cochlear implantation regarding auditory rehabilitation, quality of life, tinnitus, and stress. Laryngoscope.

[23] Noble W, Gatehouse S (2006). Effects of bilateral versus unilateral hearing aid fitting on abilities measured by the speech, spatial, and qualities of hearing scale (SSQ). Int J Audiol.

[24] R Core Team (2019). R: A language and environment for statistical computing. R Foundation for Statistical Computing, Vienna, Austria. URL https://www.R-project.org/

[25] Sorkin DL, Buchman CA (2016). Cochlear implant access in six developed countries. OtolNeurotol.

[26] Di Nardo W, Anzivino R, Giannantonio S, Schinaia L, Paludetti G (2014). The effects of cochlear implantation on quality of life in the elderly. Eur Arch Otorhinolaryngol.

